# Effect of phosphodiesterase-5 inhibitors (PDE5is) on the treatment of male infertility: A protocol for systematic review and meta-analysis

**DOI:** 10.1097/MD.0000000000018317

**Published:** 2019-12-16

**Authors:** Liang Dong, Xiaojin Zhang, Xuhong Yan, Yifeng Shen, Xujun Yu, Yulin Li

**Affiliations:** a Department of Andrology, The Reproductive & Women-Children Hospital, Chengdu University of Traditional Chinese Medicine; b Chengdu University of Traditional Chinese Medicine; c Hospital of Chengdu University of Traditional Chinese Medicine, Chengdu, Sichuan, PR China.

**Keywords:** male infertility, meta-analysis, phosphodiesterase-5 inhibitors (PDE5is), protocol, sperm, systematic review

## Abstract

**Background:**

Male infertility is a worldwide problem with limitations in the treatment. Phosphodiesterase-5 inhibitors (PDE5is) is the first choice for the treatment of erectile dysfunction (ED), but more and more studies show that it has a certain effect on male infertility in recent years. The literatures of PDE5is related to male infertility have shown inconsistent results, and there is currently no high quality of systematic review to evaluate the effects of PDE5is on semen quality in male infertility patients.

**Methods:**

The electronic databases of MEDLINE, PubMed, Web of Science, EMBASE, Clinicaltrials.org, China National Knowledge Infrastructure Database (CNKI), Wan fang Database, China Biology Medicine Database (CBM), VIP Science Technology Periodical Database, Chinese Clinical Trial Registry and Cochrane Library were retrieved. Grey literature will be searched in Open Grey. Related Randomized controlled trials (RCTs) were collected and selected before October 20, 2019. We will search English literature and Chinese literature with search terms “male infertility”, “phosphodiesterase-5 inhibitors”, “PDE5i”, “Tadalafil”, “Sildenafil”, “Vardenafil”, “Udenafil”, “Avanafil”, “semen” and “sperm”. Sperm concentration, motility and morphology, sperm DNA fragmentation index, number of per ejaculate, sperm viability and adverse events will be evaluated. RevMan 5.3 and Stata 14.0 will be used for Systematic review and Meta-analysis. This protocol reported in accordance with the Preferred Reporting Items for Systematic Reviews and Meta-Analyses Protocols (PRISMA-P) statement, and we will report the systematic review by following the PRISMA statement.

**Conclusion and dissemination:**

Efficacy and safety of PDE5is on male sperm quality in infertile men will be assessed. The results will be published in a public issue journal to provide evidence-based medical evidence for urologists and andrologists to make clinical decisions.

**Registration information:**

PROSPERO CRD42019142980

## 1 Introduction

There are approximately 200 million infertile patients worldwide, and male infertility accounts for about 50%.^[1]^ The literature suggests that many reasons can lead to male infertility.^[2]^ Phosphodiesterase-5 inhibitors (PDE5is) can block the decomposition of cyclic guanosine monophosphat (cGMP), which leads to the dilation of the blood vessels of penis, and thus enhances the activity of the nitric oxide-cGMP pathway that promotes penile erection.^[3]^ PDE5is are currently widely used in the treatment of erectile dysfunction (ED) in men with definite efficacy.^[4]^ At the same time, some studies have also found that PED5is can improve the quality of male sperm, thereby improving male fertility.^[5]^ Especially at present, there is no definite effective drug to treat male infertility, so further research on PDE5is can expand its scope of application for the benefit of male infertility patients.

At present, there has been systematic review^[6]^ to confirm the improvement effect of PDE5is on sperm quality, but the latest clinical included research is 2015. Four years has passed, we have retrieved a part of new and valuable clinical research, and the increased sample size may have some changes to the conclusion. We also urgently need to understand the specific details and aspects of PDE5is improvement in sperm quality, so as to better improve clinical evidence-based medicine decision-making for urologists and andrologists. Therefore, a systematic review and meta-analysis of this subject are carried out again.

## 2 Review objectives

The purpose of this study is to evaluate the effect of PDE5is on sperm quality of male infertility, including sperm concentration, motility and morphology, sperm DNA fragmentation index, number of per ejaculate, etc. The results will provide clinical drug decisions for urologists and andrologists.

## 3 Methods

This is a systematic review, with a meta-analysis if necessary. The data and results used in this paper are almost from published studies, and there are no ethical issues, so the approval of the ethics committee is not required.

### 3.1 Protocol and registration

This study is registered on PROSPERO. Registration number: CRD42019142980.

This protocol refers to the statement of Preferred Reporting Items for Systematic Reviews and Meta-Analyses Protocols (PRISMA-P).^[7,8]^ And we will report the systematic review in accordance with the PRISMA statement strictly.

### 3.2 Data source

#### 3.2.1 Electronic search database and approach

The electronic databases of MEDLINE, PubMed, Web of Science, EMBASE, Clinicaltrials.org, China National Knowledge Infrastructure Database (CNKI), Wan fang Database, China Biology Medicine Database (CBM), VIP Science Technology Periodical Database, Chinese Clinical Trial Registry and Cochrane Library will be retrieved. Grey literature will be searched in Open Grey. Related Randomized controlled trials (RCTs) will be collected and selected before October 20, 2019. The searching work will be done in November 2019 and updated before the systematic review has completed.

Medical Subject Heading or text key words “male infertility” or “semen” or “sperm” AND “phosphodiesterase-5 inhibitors” or “PDE5i” or “Tadalafil” or “Sildenafil” or “Vardenafil” or “Udenafil” or “Avanafil” will be used. The search strategy is adjusted to fit the different databases. Chinese form of the above terms will be used in Chinese search. A specific search example for MEDLINE is shown in Table 1.

**Table 1:**
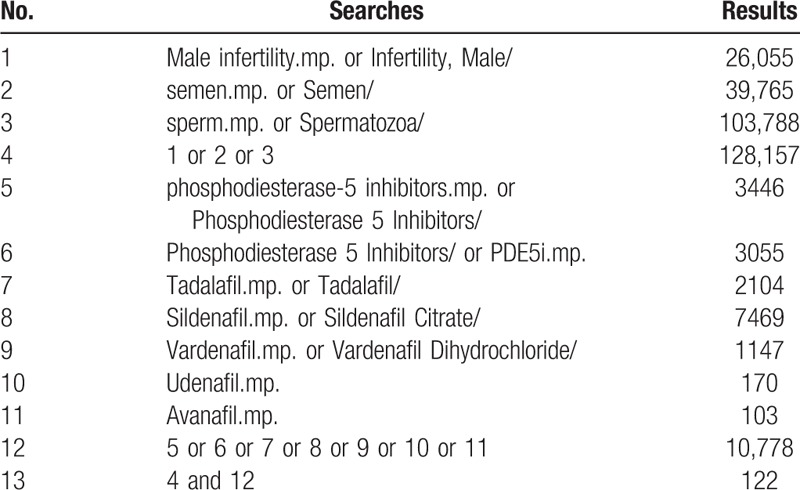
MEDLINE search strategies.

#### 3.2.2 Other sources of search

Grey literature will be retrieved through Open Grey. Acquisition of full-text documents requires library interlibrary loan and Google academic. A manual review of references in published articles will be conducted to identify other relevant studies, and references detected from existing system reviews and meta-analyses will also be retrieved.

### 3.3 Included and excluded criteria

#### 3.3.1 Study design

Only RCTs will be included in this study. Observational studies, retrospective analyses, self-controlled trials, patient series, case reports, reviews, animal studies, and laboratory in vitro studies, no data to extract, will be excluded.

#### 3.3.2 Participants

##### 3.3.2.1 Included population

Men of childbearing age who have fertility requirements and semen samples at baseline met the World Health Organization (WHO) 1999 criteria (sperm concentration of 20 million/ml (geometric mean of two samples), 50% motile sperm, 15% sperm with normal morphology, and semen volume 1.5 ml (arithmetic mean of 2 samples)^[9]^ and 2009 criteria (sperm concentration of 15 million/ml (geometric mean of two samples), 32% PR sperm, 4% sperm with normal morphology, and semen volume 1.5 ml (arithmetic mean of 2 samples).^[10]^

##### 3.3.2.2 Excluded population

Undiagnosed patients; azoospermia; infertility result from hypothalamic-pituitary-gonadal lesion, chromosomal or genetic lesion, hormone abnormality, congenital diseases, obstructive diseases.

#### 3.3.3 Interventions

Randomized, double-blind, placebo-controlled trials are identified to be the best.

Treatment group: This group was treated with PDE5i, include “Tadalafil” or “Sildenafil” or “Vardenafil” or “Udenafil” or “Avanafil”. The patients may have erectile dysfunction, but should not affect vaginal insertion, that is to say mild erectile dysfunction was accepted, not moderate and severe erectile dysfunction.

Control group: a placebo with the same appearance as the treatment group.

#### 3.3.4 Outcomes

##### 3.3.4.1 Primary outcome indicator

1) Sperm concentration: number of sperm per milliliter (×10^6^/mL).^[11]^
2) Sperm motility: including spermatozoa with activity of A and B levels or spermatozoa with forward-moving sperm in the WHO classification, provided as a percentage (%).
3) Sperm morphology: proper sperm ratio, provided as a percentage (%).^[11]^

It will be based on the results reported at the end of included studies.

##### 3.3.4.2 Secondary outcome indicators:

1) Sperm DNA fragmentation index: Sperm DNA damage was reported in the study. The detection method may be sperm chromatin structure assay (SCSA), terminal deoxyuridine nick end labelling (TUNEL) assay, Comet assay, sperm Chromatin Dispersion (SCD) assay, Acridine orange (AO) test, Aniline blue (AB) staining, Toluidine blue, Chromomycin A3 (CMA3) staining.^[12]^
2) Number per ejaculate: The total number of sperm contained in once ejaculation (×10^6^/once ejaculation).
3) Sperm viability: Proportion of all active sperm (including A, B, C or PR, NP), provided as a percentage (%).
4) Adverse events: all adverse events, including nausea, vomiting, facial flushing, increased heart rate and other adverse events.

### 3.4 Selection of studies and data extraction

Document management will be conducted by Endnote X9 software. The software will be used to filter duplicate documents first, and then delete duplicate documents by reading titles, abstracts and other relevant information.

According to the inclusion and exclusion criteria, the literature will be further screened. In this process, the controversial literature will be screened after obtaining the full text. Further detailed screening and data extraction of the documents will be carried out simultaneously by two professionally trained reviewers (Xiaojin Zhang, Liang Dong).

**Figure 1:**
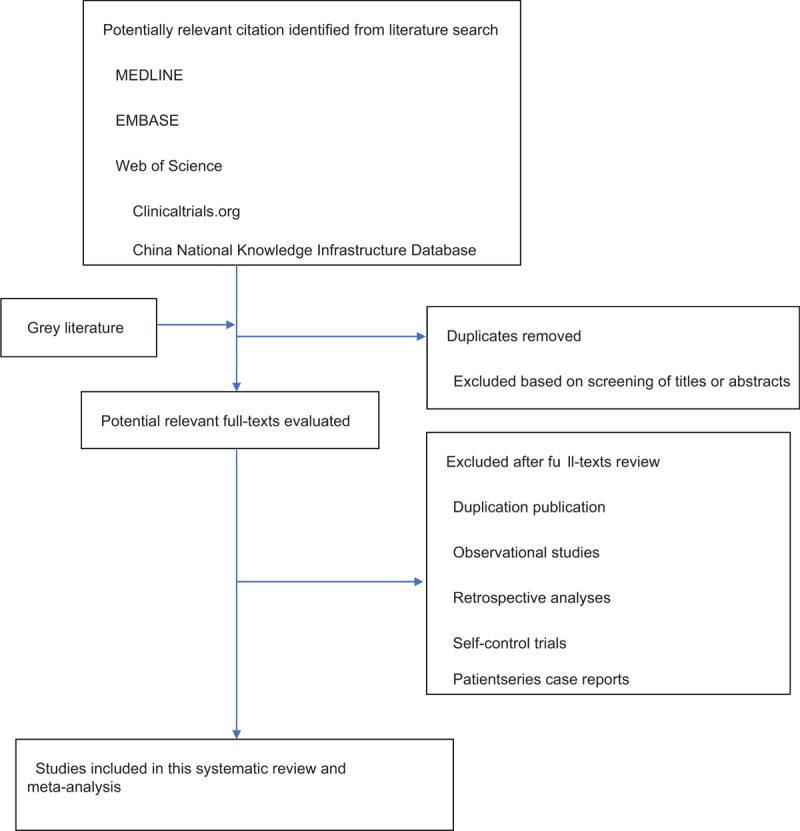
The PRISMA literature screening flow chart.

Then, the articles that meet the inclusion criteria are full-text read and re-screened. If two or more articles have repeated or staged research results, only the articles with the largest sample size, the most complete intervention and follow-up time are included. When the review team cannot confirm the repeated studies, the original study author will be contacted for judgment. The flow chart of literature screening is shown in Figure 1. All articles which comparing PDE5is with placebo intervention or sedentary RCTs (Including conference papers that can be obtained by contacting the author for the original research details) will be included.

Before the formal process of data extraction, the review group will discuss and a unified data extraction form (an excel spreadsheet) will be produced. Two review authors (Liang Dong, Xiaojin Zhang) will independently conduct data extraction exercises, extract relevant data of 3 articles, and further test the consistency of data extraction and the integrity of the table. All differences will be discussed and resolved with the third reviewer (Yulin Li). The content of data extraction is as follows.

1) General characteristics: name of first author, publishing year, study tittle, nation or country, execution time of study, e-mail or other contact information.
2) Information of studies: study design, sample size, randomized information, assignment hiding, blind method, diagnostic criteria, outcome indicators, safety indicators, statistical methods, information of outcome indicators, follow-up.
3) Information of participants: age, severity of disease, course of disease, baseline level, comorbidity, healthy condition.
4) Information of control group: The packaging, shape, taste and color of the oral drug should be consistent with that of the treatment group, and neither the researcher nor the participants could distinguish them.
5) Outcome indicators: Detailed statistics of sperm quality parameters, including sperm concentration, sperm motility, sperm morphology, sperm DNA fragmentation index, number per ejaculate, sperm viability, data of adverse events and specific information.
6) Risk of bias: random sequence generation, allocation concealment, blinding of participants and personnel, incomplete outcome data, selective reporting, other bias.^[13]^
7) Other study information: funding situation, conflict of interest.

If there are abstracts of the conference papers meeting the inclusion criteria, those RCTs will be included in the analysis based on the recommendations of Systematic Reviews section 6.2.2.4 in Cochrane Handbook.^[14]^ When necessary, the review team will contact the original research author via email to obtain the full text or relevant results. And if there are any questions or puzzles about the original research in the process of data extraction, the author will be contacted again to get specific answers.

### 3.5 Risk of bias assessment

Selection bias, performance bias, detection bias, attrition bias, reporting bias and other bias will be assessment based on the Cochrane Collaboration Network Risk Assessment Tool. Two review authors (Xianjin Zhang, Liang Dong) will independently evaluate and cross check the risk of bias. Discrepancies between review authors on the risk of bias will be resolved through discussion with a third review author (Yulin Li).

Assessment items include random sequence generation, allocation concealment, blinding of participants and personnel, blinding of outcome assessment, incomplete outcome data, selective reporting and other bias.^[13]^ Each item of bias situation includes low risk, unclear and high risk.^[13]^ Since we can’t determine the authenticity of blindness, the outcome indicators of the systematic review are relatively objective, so we define the generation of random sequence, allocation concealment and incomplete data as key domains of risk of bias evaluation. The risk of bias assessment chart of inclusion studies will be produced by using Review Manager 5.3 software.

### 3.6 Data analysis and synthesis

Descriptive analysis or narrative synthesis will be performed when there are clinical heterogeneity among the studies or when the data cannot be synthesized or results data cannot be extracted. When included trials are clinically homogeneous and the data are similar and synthesizable, a meta-analysis will be performed. Dichotomous variable will be pooled as RR and 95% confidence intervals. Continuous variable will be pooled as MD and 95% confidence intervals. We will use Cochran's Q statistic and I^2^ statistic to test heterogeneity. *P* < .10 is heterogeneous, or I^2^ > 50% is significant heterogeneity. A fixed effect model (Mantel-Haenzel method for RR and Inverse Variance for MD) will be used for I^2^ < 50%. A random effects model (D-L method) will be used when the heterogeneity is still significant after sensitivity analysis and subgroup analysis. A *P* < .05 of z test will be considered statistically significant. The meta-analysis will be generated by Review Manager 5.3 software and displayed as a forest plot, while a funnel plot will be generated to assess the risk of bias.

### 3.7 Subgroup analysis

If the data is sufficient and there is heterogeneity between studies, we will perform a subgroup analysis. Subgroup analysis will be performed according to different age, ethnicity, kind of male infertility, comorbidity, interventions, control measures, measurement methods or time of measurement.

### 3.8 Sensitivity analysis

Sensitivity analysis will be used to test the reliability and stability of the meta-analysis results, and to detect the source of heterogeneity. This can be done by excluding trials with a high risk of bias or eliminating each study individually. The meta-analysis will then be performed again and the results compared with the previous meta-analysis.^[15]^

### 3.9 Publication bias

Publication bias will be measured by using a funnel plot (by Review Manager 5.3 software), Begg test and Egger test (by Stata software 14.0).^[15]^

## 4 Discussion

Male infertility is not an independent disease. It is a result of multiple factors, idiopathic infertility accounts for 30% of male infertility.^[16]^ Many different kinds of drugs were tried to improve semen quality, including PDE5is, a drug used to be treating erectile dysfunction. Such studies have found that PDE5is can improve semen quality in somehow, with no adverse effects on sperm prtial parameter.^[5,17–20]^

PDE5is includes tadalafil, sildenafil, udinafid, avanafil and vardenafil at present, each of them has its specific characteristics. Sildenafil acts quickly and works well. Tadalafil has a long half-life period and can last 36 hours effectively. Vardenafil can be taken after drinking.^[21]^ Udinafide has clinical characteristics of relatively rapid onset and long duration of action, which is another option for daily administration of ED.^[22]^ As the second generation of PDE5is, avanafil has the fastest effect, lower incidence of drug-related side effects such as dyspepsia compared to the other PDE5is.^[23]^

Although there has been a systematic review of PDE5is improving sperm quality before,^[6]^ we believe that the new included research literature will bring more beneficial conclusions. In the other side, more detailed analysis of different drugs can also bring some challenges to this systematic review. Through this study, more detailed observation and analysis of the efficacy of PDE5is can guide physicians to use drugs more rationally and more specifically. This will be the greatest value in this systematic review.

This systematic review has the following limitations: First, as we are not good at other languages, the literatures we searched are limited to Chinese and English, which will cause certain bias. Second, different PDE5is have different characteristics and usage, so the effect on sperm quality may be different. Third, the limitation of sample size also leads to the instability of conclusion reliability. We are still looking forward to better and larger scale studies to confirm the effect of PDE5is on sperm.

## 5 Amendment

We have added a search term “Avanafil” to improve the electronic search process, so that we can more comprehensively cover the drugs contained in PDE5is. But the drug Avanafil was not mentioned when this systematic review was registered.

## Author contributions

**Research design and concept:** Liang Dong, Xiaojin Zhang, Yulin Li

**Program management:** Liang Dong, Xuhong Yan, Yulin Li

PDE5is related expertise is provided: Liang Dong, Yulin Li

**Data management:** Liang Dong, Yulin Li, Xiaojin Zhang

**Methodology:** Liang Dong, Yulin Li, Yifeng Shen

**Software:** Yulin Li, Yifeng Shen

**Draft writing:** Liang Dong, Xiaojin Zhang, Yulin Li

**Manuscript modification and editing**: Liang Dong, Xuhong Yan, Yulin Li

**Review the manuscript and approve the release**: Liang Dong, Xujun Yu, Yulin Li

**Resource:** Xujun Yu, Yulin Li

**Conceptualization:** Xiaojin Zhang.

**Data curation:** Xiaojin Zhang.

**Writing – original draft:** Xiaojin Zhang.

Liang Dong orcid: 0000-0002-6628-2106.
